# Influence of patterned sapphire substrates with different symmetry on the light output power of InGaN-based LEDs

**DOI:** 10.1186/1556-276X-9-596

**Published:** 2014-11-03

**Authors:** Yao-Hong You, Vin-Cent Su, Ti-En Ho, Bo-Wen Lin, Ming-Lun Lee, Atanu Das, Wen-Ching Hsu, Chieh-Hsiung Kuan, Ray-Ming Lin

**Affiliations:** 1 Graduate Institute of Electronic Engineering and Department of Electrical Engineering, National Taiwan University, No. 1, Roosevelt Road Section 4, Daan District, Taipei 10617, Taiwan; 2 Graduate Institute of Electronic Engineering and Green Technology Research Center, Chang Gung University, 259 Wen-Hwa 1st Road, Kwei-Shan, Tao-Yuan 333, Taiwan; 3 Department of Materials Science and Engineering, National Chiao Tung University, No. 1001, Daxue Road, East District, Hsinchu 300, Taiwan; 4 Sino-American Silicon Products Incorporated, No. 8, Industrial East Road 2, Science-Based Industrial Park, Hsinchu 300, Taiwan

**Keywords:** Light-emitting diodes, GaN, Patterned sapphire substrates, Quantum-confined Stark effect

## Abstract

This paper aims to investigate the light output power (LOP) of InGaN-based light-emitting diodes (LEDs) grown on patterned sapphire substrates (PSSs) with different symmetry. The GaN epitaxial layers grown on the hexagonal lattice arrangement PSS (HLAPSS) have a lower compressive strain than the ones grown on the square lattice arrangement PSS (SLAPSS). The quantum-confined Stark effect (QCSE) is also affected by the residual compressive strain. Based on the experimentally measured data and the ray tracing simulation results, the InGaN-based LED with the HLAPSS has a higher LOP than the one with the SLAPSS due to the weaker QCSE within multiple-quantum wells (MQWs).

## Background

In recent years, InGaN-based light-emitting diodes (LEDs) are widely used for applications in the backlight of flat-panel displays and solid-state lighting [1]. In order to compete with conventional lighting sources and realize the ultimate lamp, the external quantum efficiency (EQE) of InGaN-based LEDs must be enhanced. The EQE of InGaN-based LEDs is determined by internal quantum efficiency (IQE) and light extraction efficiency (LEE). Many significant methods have been proposed in the literatures for improving the EQE of InGaN-based LEDs, such as patterned sapphire substrates (PSSs) [2-6], epitaxial lateral overgrowth (ELOG) [7,8], surface structure [9-11], semi/non-polar quantum wells (QWs) [12-15], and so on. However, among these technologies, the PSS method has attracted considerable attention because of its ability to improve both IQE and LEE. While implementing the InGaN-based LEDs on PSSs with various periodic patterns, structural parameters of PSSs should be taken into consideration cautiously. The previous published articles have shown that the light output power (LOP) of InGaN-based LEDs is dependent on the configuration of these parameters including spacing [16], slanted angle [17], shape [18], height [19], and density [20]. Nevertheless, studies concerning the effect of PSSs with different symmetry on the LOP of InGaN-based LEDs were still limited.

In this paper, the effect between the LOP and the InGaN-based LEDs grown on PSSs with different symmetry is investigated in detail through simulation and measurements. The different symmetry of PSS used for experimentation include square lattice arrangement (SLA) and hexagonal lattice arrangement (HLA).

## Methods

**Figure 1:**
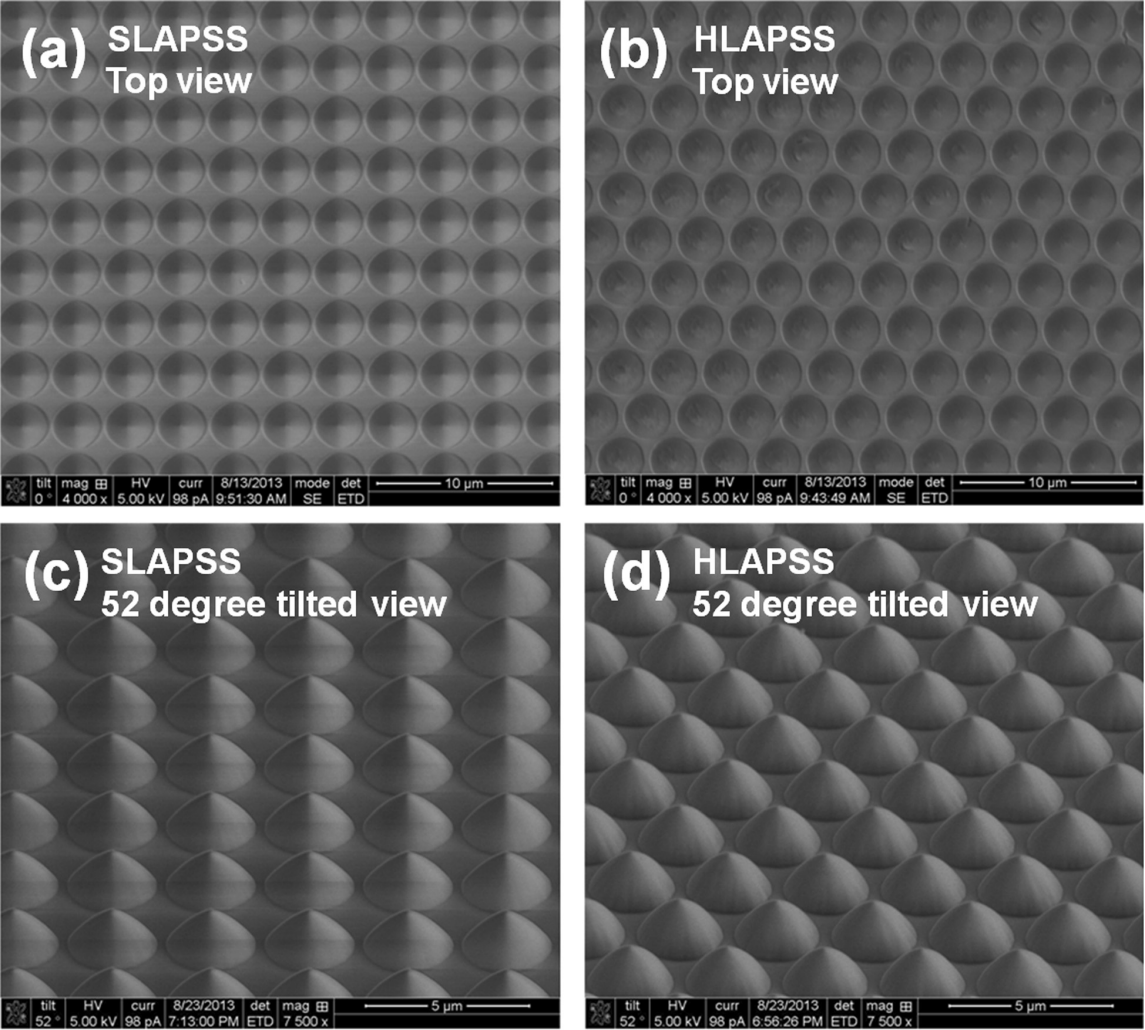
**The SEM images of PSSs with different symmetry. (a, b)** Top surface views of SLAPSS and HLAPSS. **(c, d)** Fifty-two-degree tilted views of SLAPSS and HLAPSS.

The SLAPSS and HLAPSS were fabricated with photolithography and dry etching technology. The photoresist pattern was transferred to the substrate directly by inductively coupled plasma reactive ion etching (ICP-RIE). The post patterns with the diameter of 2.65 μm, height of 1.6 μm, and periodicity of 3 μm were fabricated on a 2-in. c-plane sapphire substrate. The surface morphology, periodicity, height, and diameter of the accomplished PSSs were examined by FEI Dual-Beam NOVA 600i Focused Ion Beam (FEI, Hillsboro, OR, USA) as shown in Figure 1.

After the cleaning process, the InGaN-based LED samples were grown on the PSSs with Taiyo Nippon Sanso SR2000 (Taiyo Nippon Sanso, Dalian, China) atmospheric pressure metal organic chemical vapor deposition (AP-MOCVD) under a three-flow gas injection. Prior to the growth, substrates were thermally baked at 1,180°C in hydrogen gas to remove surface contamination. The InGaN-based LED structures were initially grown on the PSSs, and their structure consists of a 25-nm-thick low-temperature GaN nucleation layer, a 2.5-μm-thick unintentionally doped GaN buffer layer (grown at 1,180°C), and a 3-μm-thick n-GaN layer, using SiH_4_ as the n-type dopant. Then, five pairs of InGaN/GaN multiple-quantum wells (MQWs) having a 2.9-nm-thick InGaN well and an 11-nm-thick GaN barrier (grown at 800°C and 850°C, respectively) were deposited, followed by a 20-nm-thick p-AlGaN electron-blocking layer and a 120-nm-thick p-GaN layer, using Cp_2_Mg as a p-type dopant. The InGaN-based LEDs with a conventional sapphire substrate (CSS), SLAPSS, and HLAPSS were grown under the same growth condition.

**Figure 2:**
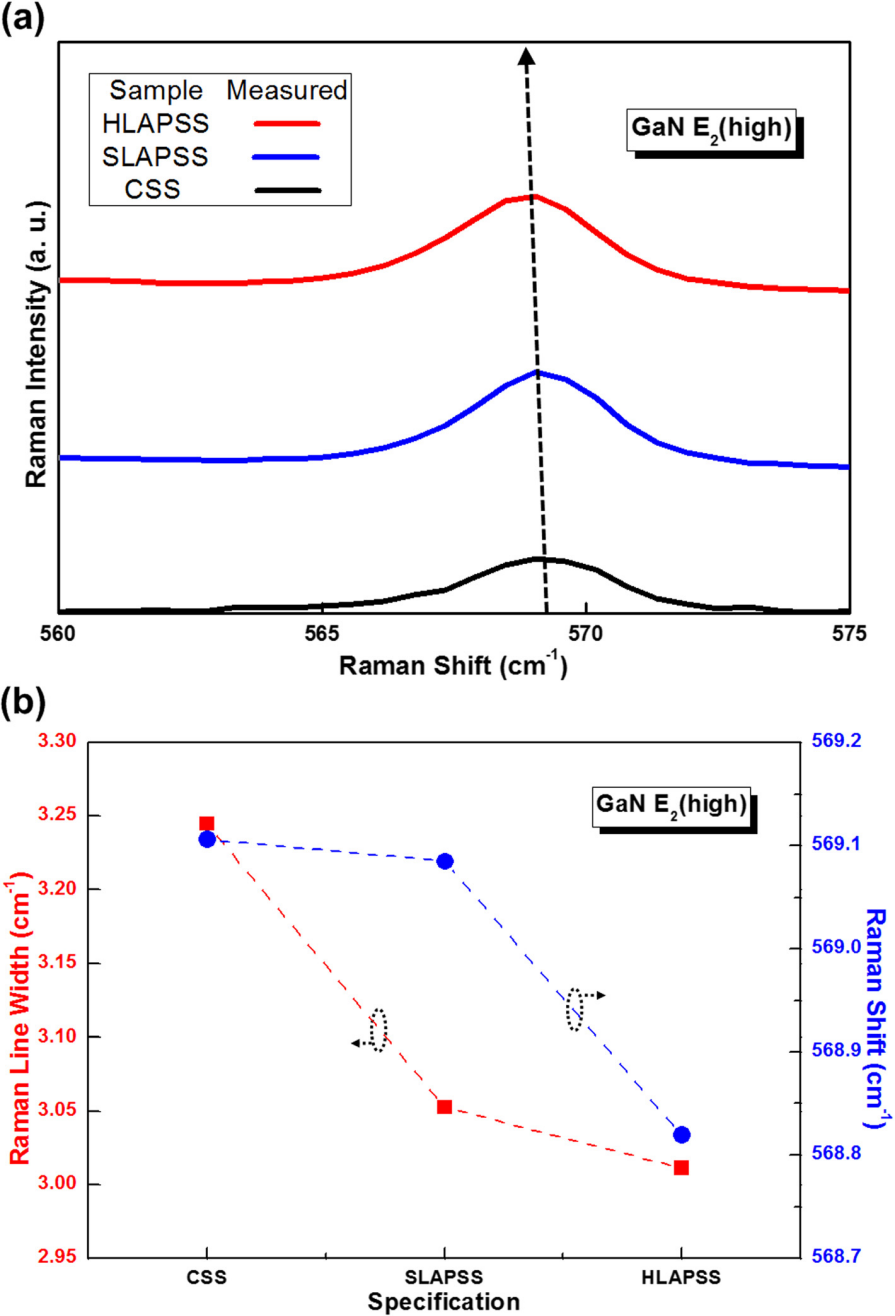
**Raman results for the InGaN-based LEDs on the CSS and PSSs. (a)** The room temperature Raman spectra and **(b)** the corresponding room temperature Raman shift and Raman line width.

To gain insight into the correlation between the PSSs with different symmetry and the strain variation in the GaN epitaxial layers, the micro-Raman measurement is required. Figure 2a shows the room temperature Raman spectra, and Figure 2b indicates the associated Raman shift and line width of GaN E_2_(high) mode of the InGaN-based LEDs having the CSS, SLAPSS, and HLAPSS. The Raman shifts of the E_2_(high) mode of InGaN-based LEDs grown on the CSS, SLAPSS, and HLAPSS are 569.11, 569.08, and 568.82 cm^-1^, respectively. Since the literature [21] demonstrated that the E_2_(high) phonon frequency of a perfect GaN is 567.6 cm^-1^ at room temperature measurement and the residual compressive strain can be calculated through the measured E_2_(high) mode Raman shift [22], the associated residual compressive strain is calculated to be -1.22 × 10^-3^ for the InGaN-based LEDs grown on the CSS. The other calculated values of the residual compressive strain are -1.21 × 10^-3^ and -1.07 × 10^-3^ for the InGaN-based LEDs having the SLAPSS and HLAPSS, respectively. This reveals that the InGaN-based LED grown on the HLAPSS has the lowest residual compressive strain. The smallest Raman line width of the sample with HLAPSS is also shown in the figure. These results may imply that the growths of InGaN-based LED on the HLAPSS can improve the bulk GaN crystalline quality through the relaxation of the residual compressive strain as a result of the higher symmetry of HLAPSS.

**Figure 3:**
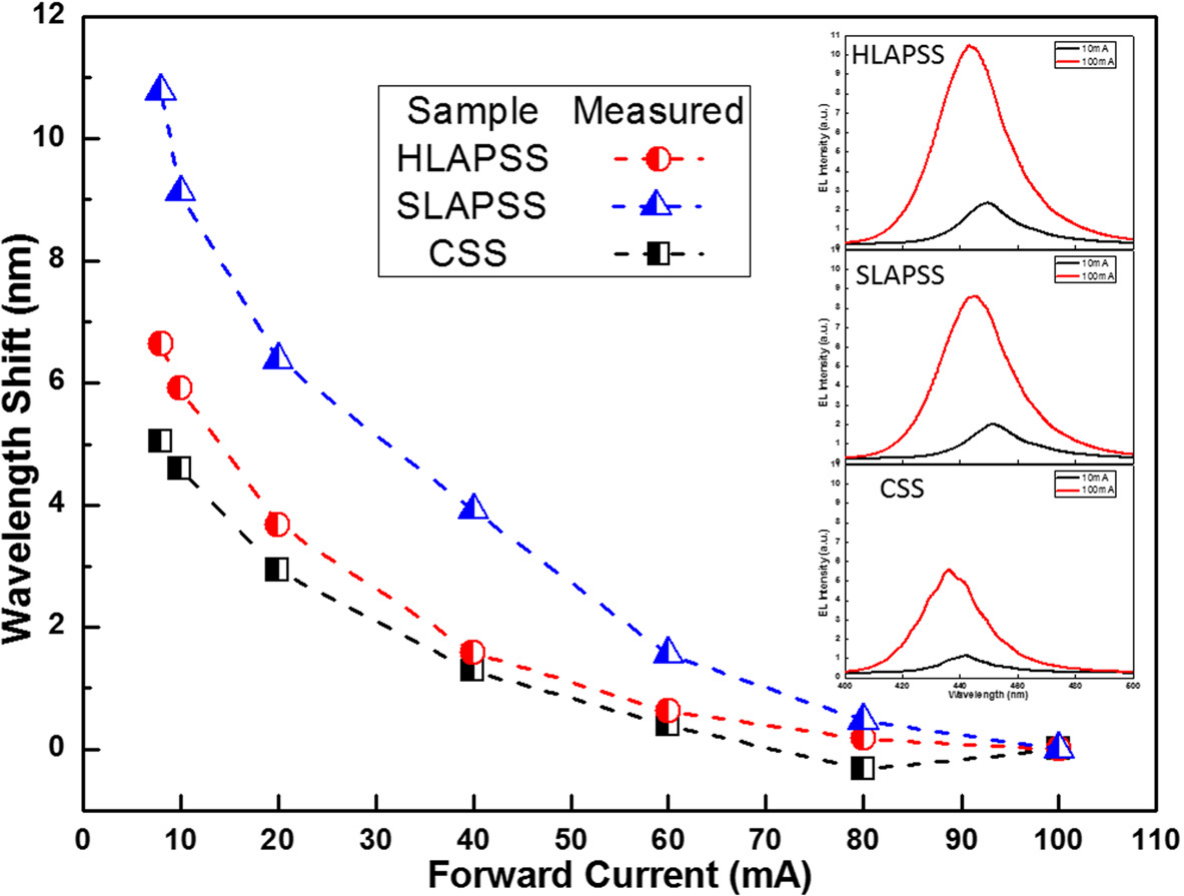
**Blueshift phenomenon of the InGaN-based LEDs having the CSS, SLAPSS, and HLAPSS.** Peak wavelength shift versus injection current of the InGaN-based LEDs grown on CSS and PSSs with different symmetry.

Based on the previous study published by our group [23], the smaller residual compressive strain can result in a weaker quantum-confined Stark effect (QCSE) in MQWs, which enhanced the LOP of InGaN-based LEDs. Therefore, the effect of InGaN-based LEDs having the PSSs with different symmetry on the QCSE was further elucidated by utilizing the excitation current-dependent electroluminescence (EL) measurement. Figure 3 shows the peak wavelength shifts of the EL spectrum under different injection currents for the InGaN-based LEDs with the CSS, SLAPSS, and HLAPSS. The insets of Figure 3 focus on the EL spectra of InGaN-based LEDs having the CSS, SLAPSS, and HLAPSS at the injection currents of 10 and 100 mA, respectively. As shown in the figure, the shifts in EL peak wavelength between 10 and 100 mA forward current for the InGaN-based LEDs with SLAPSS and HLAPSS are 10.8 and 6.6 nm, respectively. Furthermore, the EL peak wavelengths of the InGaN-based LEDs with SLAPSS and HLAPSS always appear blueshift with respect to the reference one as shown in the insets of Figure 3. Therefore, the InGaN-based LED grown on the HLAPSS has the weaker QCSE than the one that was grown on the SLAPSS. Consequently, in contrast with the InGaN-based LED grown on the SLAPSS, the LED with the HLAPSS demonstrates a stronger confinement of electrons and holes, leading to a large overlap between electron and hole wave functions. Moreover, the blueshift of the EL peak for InGaN-based LED with CSS is the smallest due to the lower indium (In) composition of QWs in InGaN-based LED with CSS [24].

## Results and discussion

**Figure 4:**
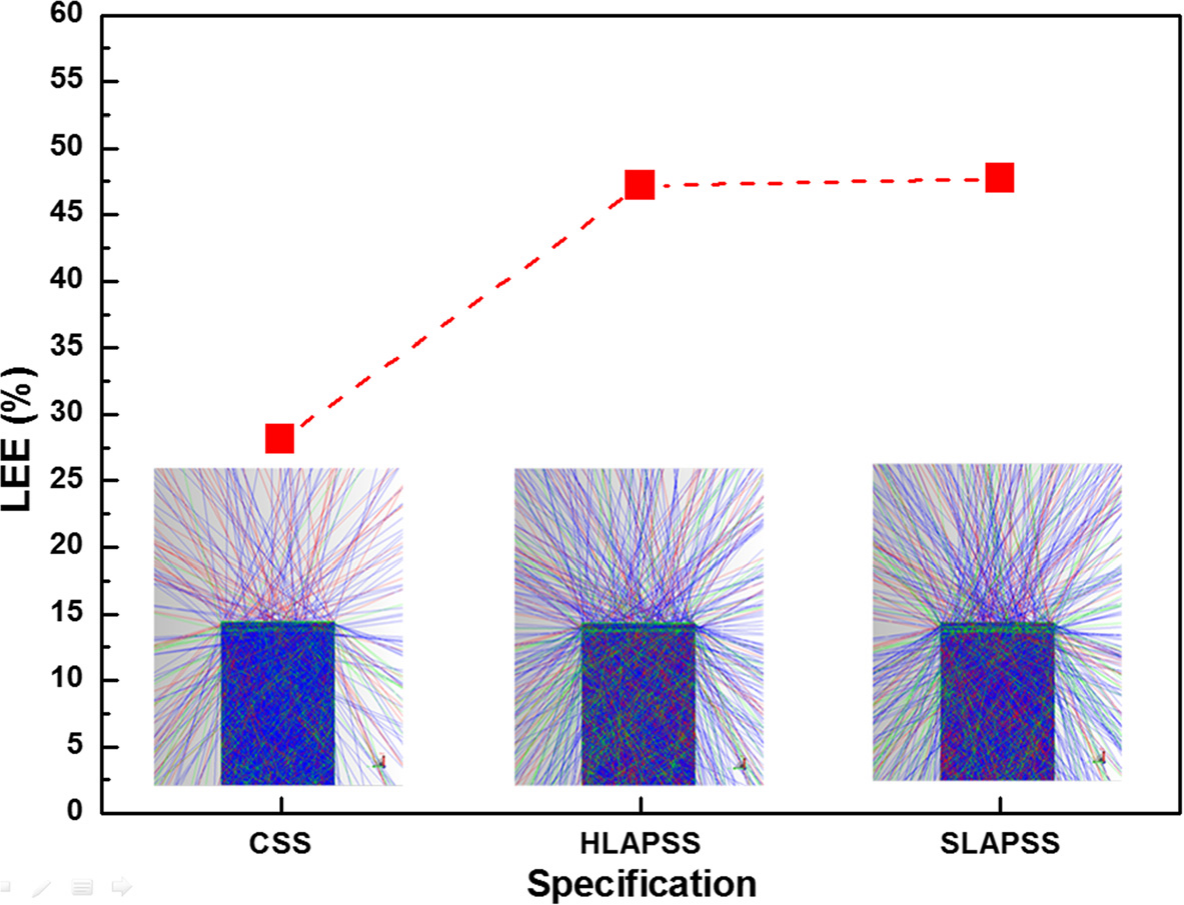
**TracePro ray tracing results for the InGaN-based LEDs having the CSS, HLAPSS, and SLAPSS.** The associated LEE with different samples is evaluated from the bare LED simulation results, and the insets show the cross-sectional ray tracing image.

Based on the experimental results of Raman and excitation current-dependent EL measurement, it can be determined that the growths of the InGaN-based LED on the higher symmetry of HLAPSS can acquire a better crystalline quality. The superiority of this structure emerges from the relieved residual compressive strain of GaN epitaxial layers, which is accompanied by the reduction of polarization fields within the MQWs along with weaker QCSE. The abatement of QCSE within the MQWs can increase overlap between electron and hole wave functions and consequently result in a stronger radiative recombination rate.To obtain the LEE contribution of PSSs with different symmetry, a TracePro (Lambda Research Corporation, Littleton, MA, USA) ray tracing simulation was used to calculate the LEE of InGaN-based LEDs. Figure 4 shows the correlated LEE of InGaN-based LEDs having the CSS and PSSs with different symmetry, and the inserted figures show the cross-sectional ray tracing image. The results reveal that the InGaN-based LEDs grown on SLAPSS and HLAPSS have the larger LEE than the one grown on the CSS due to the light scattering effect from PSSs. In addition, it is worth noting that the LEE of InGaN-based LEDs with the SLAPSS and HLAPSS are almost the same. This may be ascribed to the structural dimension, which is larger than the optical wavelength in the material. As a result, the higher symmetry of PSSs can only slightly influence the LEE.

**Figure 5:**
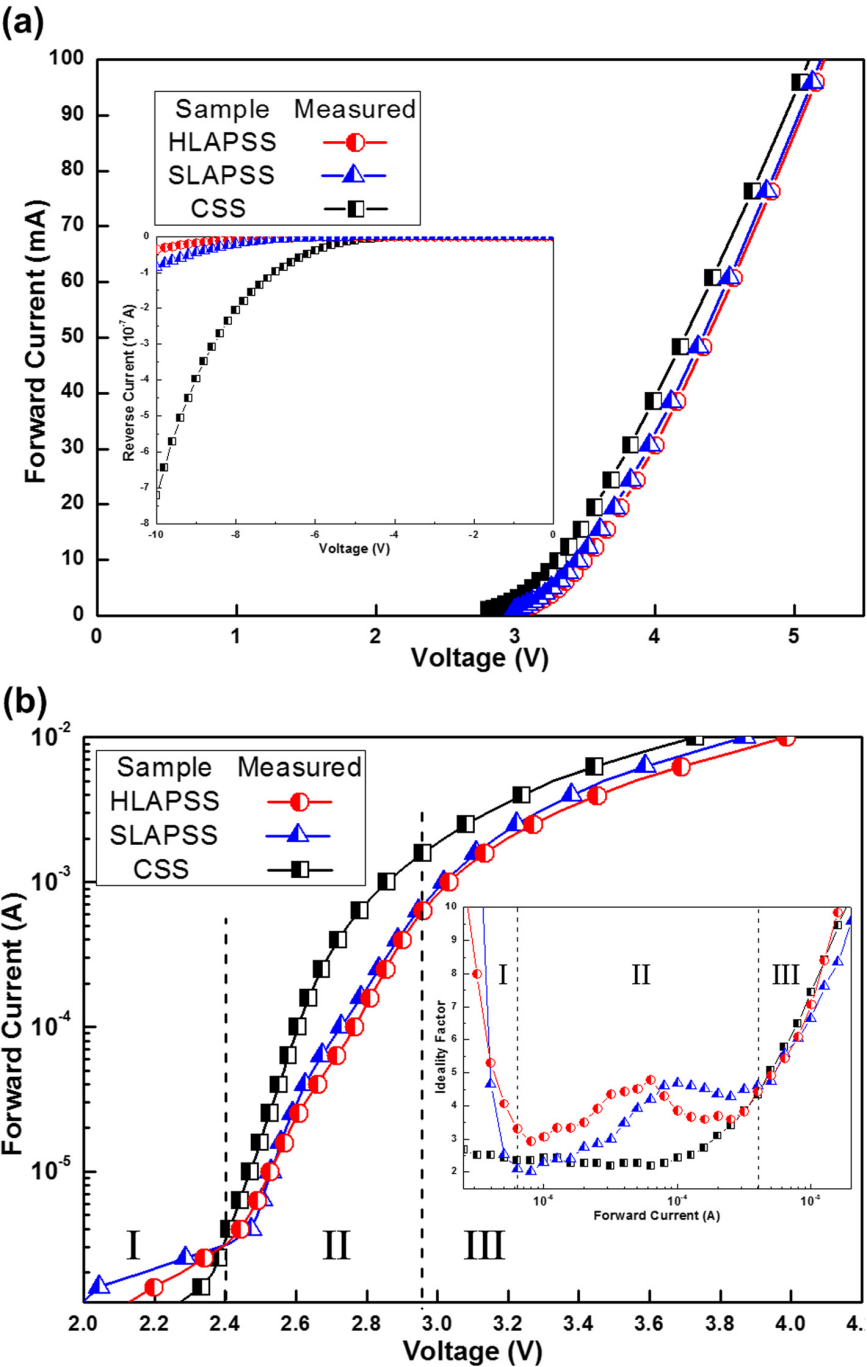
**Electrical characteristics of InGaN-based LEDs having the CSS, HLAPSS, and SLAPSS. (a)** Forward *I-V* characteristics and reverse *I-V* characteristics and **(b)** low-current *I-V* characteristics and ideality factor comparison.

**Figure 6:**
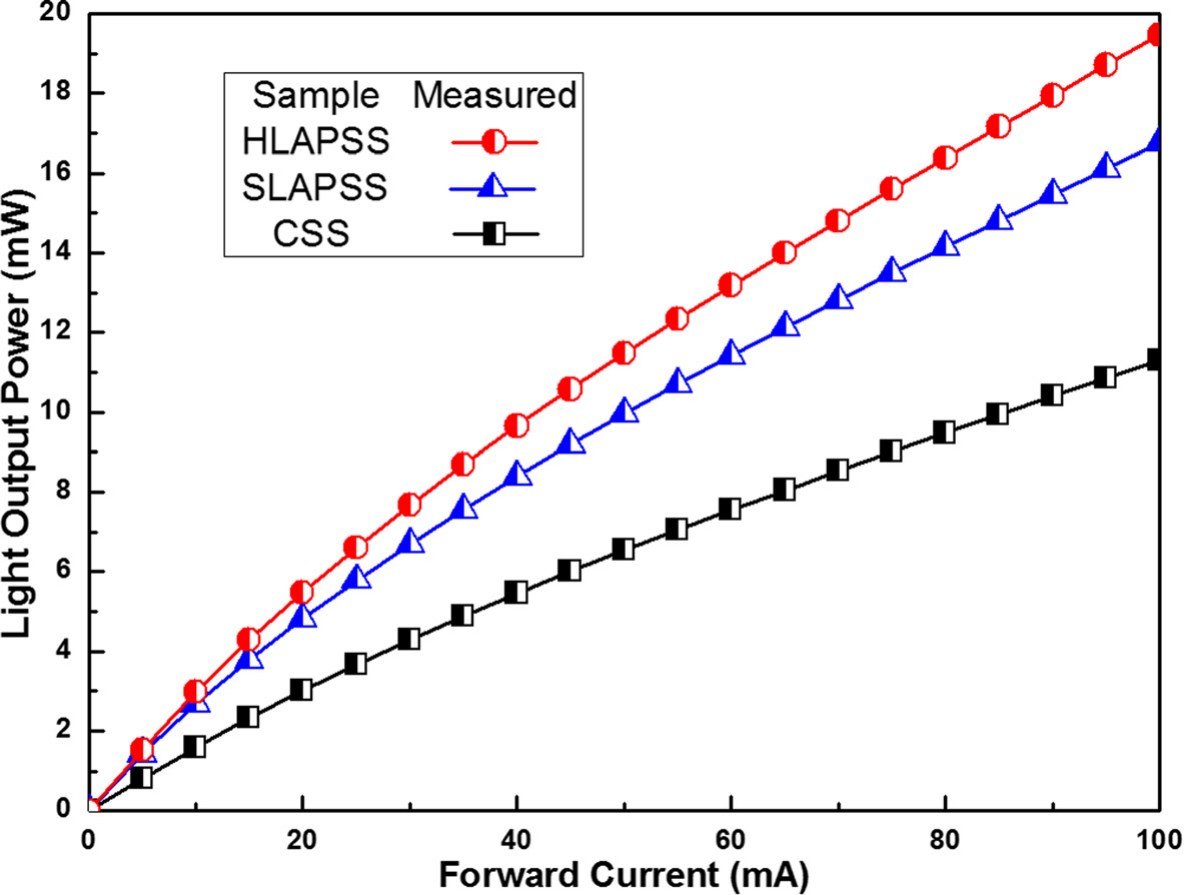
**The LOP as a function of the forward current.** The LOP of InGaN-based LEDs grown on CSS, SLAPSS, and HLAPSS is plotted as a function of the forward current.

Figure 5a shows the current-voltage (*I-V*) characteristics of the InGaN-based LEDs that are fabricated with a standard 250 × 575-μm^2^ LED die processed in the industry. The forward voltages of the InGaN-based LEDs grown on the CSS, SLAPSS, and HLAPSS at an injection current of 20 mA are 3.57, 3.71, and 3.75 V, respectively. The behaviors of the forward current versus voltage curves for the InGaN-based LEDs grown on the SLAPSS and HLAPSS are pretty similar even under a high injection current. The reverse leakage current of InGaN-based LEDs is shown in the inset of Figure 5. The reverse currents at a voltage of -10 V for the InGaN-based LEDs grown on the CSS, SLAPSS, and HLAPSS are -7.2 × 10^-7^, -8.6 × 10^-8^, and -3.5 × 10^-8^ A, respectively. In comparison to the InGaN-based LED grown on the CSS, the samples grown on the SLAPSS and HLAPSS have a lower leakage current due to the better crystalline quality of GaN epitaxial layers. This outcome agrees with the results that we have previously discussed. Figure 5b shows the 0.01- to 10-mA *I-V* comparison in a semi-log plot, and the corresponding ideality factors are also provided in the inset of Figure 5b. On closer inspection of the *I-V* curve, there are generally three different domains observed. Firstly, in the low-current domain (*I* ≤0.003 mA, domain I), the ideality factor is very high due to shunt resistance, and the ideality factor of InGaN-based LEDs with SLAPSS and HLAPSS decreases from 10 to 2.9 and 1.9, respectively, when the current increases. Secondly, in the high-current domain (*I* > 10 mA, domain III), the ideality factor is influenced by the series resistance, and the ideality factor rises with increasing current. Thirdly, in the intermediate domain (0.003 mA < *I* ≤10 mA, domain II), the impact of shunt and series resistance could be neglected, and the ideality factor gives insight into the *I-V* characteristics of the junction itself. The lowest values of ideality factors are 2.2, 2.0, and 2.9 for the InGaN-based LEDs with CSS, SLAPSS, and HLAPSS, respectively.Figure 6 presents the LOP versus forward current. The LOP values are 3.01, 4.83, and 5.48 mW for the InGaN-based LEDs grown on the CSS, SLAPSS, and HLAPSS at a current of 20 mA, respectively. Compared with the InGaN-based LED grown on the CSS at an injection current of 20 mA, the enhanced LOP values of the samples grown on the SLAPSS and HLAPSS are by up to 60% and 82%, respectively. Furthermore, the InGaN-based LED with the HLAPSS has a higher LOP than the one with the SLAPSS. According to the above simulation results which show the similar LEE of InGaN-based LEDs grown on SLAPSS and HLAPSS, the stronger LOP of the sample with HLAPSS can also be contributed from the lower QCSE acquired by the smaller residual compressive strain in the GaN epitaxial layers.

**Figure 7:**
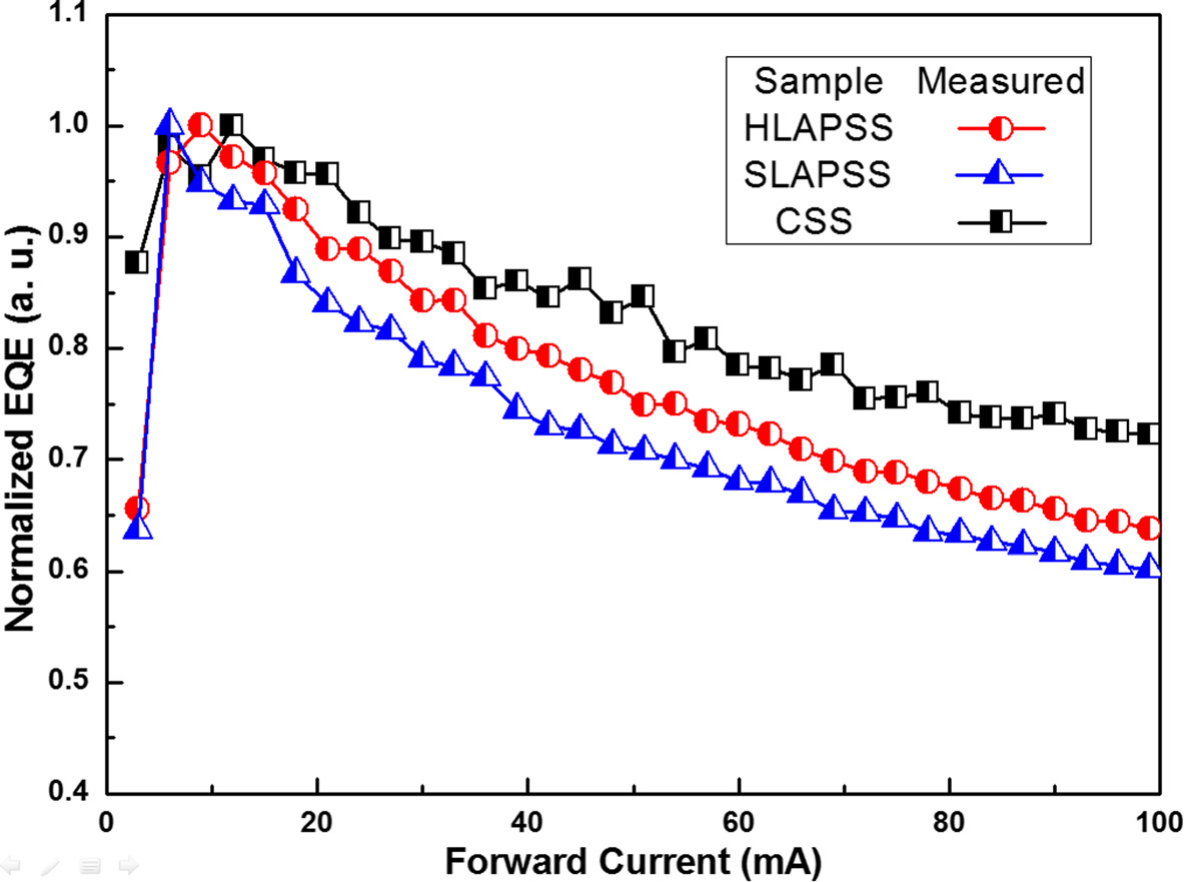
**The normalized EQE as a function of the forward current.** The normalized EQE of InGaN-based LEDs grown on CSS, SLAPSS, and HLAPSS is plotted as a function of the forward current.

Figure 7 reflects the associated normalized EQE versus the forward current of the InGaN-based LEDs having the CSS, SLAPSS, and HLAPSS. To prevent self-heating effect, the correlated efficiency droop of the devices was observed with the pulsed-mode measurement. The efficiency droop of the InGaN-based LEDs grown on the SLAPSS and HLAPSS are 64% and 60% at an injection current of 100 mA, respectively. The smaller efficiency droop is the result of the weaker QCSE within the MQWs from the use of HLAPSS. Furthermore, the InGaN-based LED grown on the CSS has the lowest efficiency droop, which is consistent with the previously discussed result of the smallest blueshift of the EL peak from the lower In composition of MQWs [25].

## Conclusions

In this paper, the superiority of InGaN-based LEDs on the HLAPSS is demonstrated. It relaxes compressive strain in the GaN epitaxial layer more than the ones on the SLAPSS. With the relaxation in compressive strain, the reduction of QCSE is observed due to the lower lattice mismatch in the InGaN/GaN MQWs. As a result, the mitigation of the efficiency droop for the InGaN-based LED on the HLAPSS occurs. Furthermore, the LEE of InGaN-based LEDs on SLAPSS and HLAPSS appeared similar from the ray tracing simulation. In comparison to the InGaN-based LED grown on the CSS at an injection current of 20 mA, the increased LOP value of the samples grown on the SLAPSS and HLAPSS is reported to be 60% and 82%, respectively.

## Competing interests

The authors declare that they have no competing interests.

## Authors’ contributions

YHY carried out the experiments and drafted the manuscript. VCS participated in the manuscript drafting and provided constructive opinions in this review paper. TEH carried out the measurements. BWL, WCH, and CHK participated in its design and coordination. MLL and AD participated in the manuscript drafting. RML conceived the study, participated in its design and coordination, and helped to draft the manuscript. All authors read and approved the final manuscript.
